# Use of geopolymers as tunable and sustained silver ion release mediums

**DOI:** 10.1038/s41598-024-59310-1

**Published:** 2024-04-13

**Authors:** Ilknur Kara

**Affiliations:** Department of Elementary Education, Faculty of Education, Anadolu University, Eskisehir, Turkey

**Keywords:** Geopolymer, Silver ion, Precipitation, Sustained release, Tunable, Antibacterial, Environmental sciences, Chemistry, Engineering, Materials science

## Abstract

Silver was incorporated up to 3.4% (w/w) into the geopolymer structure via precipitation as Ag_2_O by dispersing the geopolymer powder in an aqueous solution of AgNO_3_. The precipitates were mainly located in the fine pores within the nanoparticles of the geopolymer network. The fine pores enabled the formation of very fine precipitates, mainly between 2 and 5 nm. The silver-incorporated geopolymer was found to have a sustained Ag^+^ release that can be tuned down by a thermal treatment, e.g., calcination. The Ag^+^ release amount could be reduced by about 30-fold after calcination at 850 °C. Calcination reduces the specific surface area, causes shrinkage, and makes the geopolymer structure less pervious. The size of the precipitates remains stable even up to 1050 °C, despite a large amount of sintering-related shrinkage. These results suggest that geopolymers could be a tunable Ag^+^ source for various antibacterial applications.

## Introduction

Silver compounds have been known to have antibacterial properties for many years, and particularly silver nanoparticles are increasingly being used to remove bacteria from consumer products, medicine, and polluted waters^1–6^. Xiu et al.^7^ showed that the antibacterial effect of silver nanoparticles is not “particle-specific” and is due to silver ions (Ag^+^) produced by the oxidation of silver nanoparticles since they did not observe any antibacterial effect under anaerobic conditions under which silver (Ag^0^) oxidation is prevented. Other researchers have also confirmed that the antibacterial effect of silver nanoparticles is related to the release of Ag^+^ from them^8^. In this respect, parameters affecting the antibacterial response of silver nanoparticles, such as particle size, shape, aggregation behavior, and/or type of coating, are related to their effect on Ag^+^ release behavior. For instance, an increased antibacterial response of smaller-sized nanoparticles under the same conditions is simply due to increased Ag^+^ release because of the higher specific surface area of the smaller particles^9^.

Silver nanoparticles tend to aggregate, particularly in media with a high electrolyte content, which reduces their antibacterial activities^10^. In addition, there is a growing concern about the possible risks associated with silver nanoparticles for humans and the environment, as is the case for many other types of nanoparticles^11–13^. To avoid their strong aggregation tendency and improve antibacterial efficiency for water filtration while reducing associated health hazards, silver nanoparticles were fixed to various carriers including glass fiber^14^, rice husk ash^15^, porous ceramic filters^16,17^ and organic hollow fiber membrane^18^. Nevertheless, there is always a risk of detachment of silver nanoparticles from the carriers and their release into the aqueous environment. Wang et al.^4^ showed that silver nanoparticles detach easily from silica surfaces when shaken vigorously.

Considering that the cause of antibacterial action is Ag^+^, zeolites have been researched as effective Ag^+^ suppliers instead of silver nanoparticles. Zeolites are crystalline alkali alumina silicates with a framework structure containing mesoporosity. Alkali ions in the zeolite structure can easily be exchanged by other cations including Ag^+^ and silver uptake over 20% (w/w) is easily possible^19^. Such a high loading of Ag^+^ and its mesoporous structure makes zeolites an effective and rapid source of a large amount of Ag^+^, killing bacteria within minutes. Nanosized zeolite particles are more effective in Ag^+^ release than their micron-sized counterparts because of the shorter diffusion lengths in the nanosized zeolites^19,20^. While a large amount of Ag^+^ availability within minutes may be beneficial for some applications (e.g. medical), it may not be desirable for other applications (e.g. water filtration) where excessive Ag^+^ release may be harmful to the environment and safety.

Knowing that zeolites are excellent for fast Ag^+^ supply in large quantities, controlling Ag^+^ release rate, however, may be difficult due to their fast release behavior. To control the Ag^+^ release rate and eliminate the risk of silver nanoparticle detachment from the various carriers mentioned above, studies have been conducted on the coating of silver nanoparticles with various oxides using chemical methods. Mesoporous silica shell coating on silver nanoparticles developed by Liong et al.^21^ showed a reduced oxidation rate of the silver nanoparticles and thus reduced Ag^+^ release, resulting in long-term antibacterial properties. Gao et al.^22^ developed Fe_2_O_3_-coated silver nanoparticles attached to graphene oxide with good long-term antibacterial properties because of the reduced oxidation rate of silver nanoparticles in the presence of graphene oxide. Carbon^23^ and titania^24^ were also deposited on silver nanoparticles for the same purpose. Wang et al.^4^ further developed a novel silver-based disinfectant with a double-layer core–shell structure consisting of a superparamagnetic Fe_3_O_4_ nanosphere core, dense SiO_2_ inner shell, silver nanoparticles, and mesoporous SiO_2_ outer shell. While the superparamagnetic Fe_3_O_4_ nanosphere core ensured its good dispersity in water and allowed its easy magnetic separation after treatment, the dense SiO_2_ inner shell protected the Fe_3_O_4_ nanosphere core and allowed a good loading of silver nanoparticles. The outermost mesoporous SiO_2_ layer effectively protected the silver nanoparticles from detachment, and its mesoporous channels resulted in lower silver oxidation and dissolution for the controlled release of Ag^+^ ions.

Although the core–shell approach to avoid silver nanoparticle detachment into the environment and to enable the controlled release of Ag^+^ with associated long-lasting antibacterial effect was successful, the preparation of such multilayer nanopowders involves extensive chemical processes that may be costly, particularly for water treatment applications. Another possible route to obtaining controlled Ag^+^ release for lasting antibacterial effects could be to use geopolymers. Geopolymers are amorphous alkali alumina silicates obtained by polymerizing SiO_4_ and AlO_4_^−^ tetrahedral units (silates) linked by oxygen atoms, as defined by Davidovits^25^. The negative charge on the AlO_4_^−^ group is balanced by alkali cations. They have a large amount of structural porosity (over 30% vol) between 2 and 50 nm^26^ with good mechanical and chemical stability^27^. Similar to zeolites, charge-balancing alkali ions could be exchanged by other ions, including Ag^+^^28^, or silver-containing compounds could be precipitated in the geopolymer matrix during preparation^29^. O’Connor et al.^28^ demonstrated a complete exchange of Na^+^ with Ag^+^ and consequent antibacterial activity of Ag^+^-containing geopolymer. Luukkonen et al.^29^ precipitated Ag_2_O by adding AgNO_3_ to the fresh geopolymer paste, trapping metal ions in the geopolymer structure. The incorporation of silver nanoparticles into geopolymer paste during synthesis either on their own or with a carrier such as bentonite^5^ or silica^30^ was also studied. Bhuyan et al.^31^ prepared porous geopolymer filters by the direct foaming method and filled the pores with a colloidal silver solution. Depending on the method of silver incorporation into the geopolymer structure, Ag^+^ release behavior changes, which would affect its antibacterial property^5,29^.

Unlike zeolites and core–shell nanoparticles, the formation of a rigid network structure during synthesis enables geopolymers to be formed into various rigid shapes, such as filters, by foaming^5,29,32^ and 3D printing^29,32^ or they can be used in packed granule form^29^ or as fine powders. In this respect, geopolymers containing silver compounds could be versatile materials for various antibacterial applications, including water filtration, consumer products, and medicine. Some of these applications may require fast Ag^+^ release as a quick response to bacteria, whereas others may require controlled and sustained Ag^+^ release to have antibacterial effects without environmental hazards such as in water disinfection. Therefore, this study aims to investigate silver incorporation into the geopolymer structure and its Ag^+^ release behavior and to modify it by thermal treatment to achieve tunable, long-lasting Ag^+^ release behavior. It is known that geopolymers are thermally stable materials and that thermal treatment does not deteriorate the integrity and strength of even porous geopolymers with over 70% porosity^33^.

## Experimental

### Geopolymer synthesis

The geopolymer used in this study was synthesized using metakaolin (Metastar 501, Imerys, USA), sodium silicate solution (Bé° = 43, molar ratio SiO_2_/Na_2_O = 2.1, Koruma Klor, Izmit/Turkiye) and NaOH pellets (99%, Koruma Klor, Izmit/Turkey). The geopolymer composition with molar ratios of Na_2_O/SiO_2_ = 0.22, SiO_2_/Al_2_O_3_ = 3.2, Na_2_O/Al_2_O_3_ = 0.7, and H_2_O/Na_2_O = 13.8 was used. To prepare a batch of 300 g of the geopolymer; firstly, NaOH pellets were dissolved in sodium silicate solution by mechanical mixing (IKA® EUROSTAR 20 digital, Staufen/Germany) using an X mm diameter propeller at 500 rpm for 15 min. Then, the metakaolin powder (D_50_ = 1 µm) with a composition of 51.6% (w/w) SiO_2_, 45.0% (w/w) Al_2_O_3_, 1.9% (w/w) impurities, and 1.4% (w/w) loss-on-ignition value was poured into the solution in 5 min. under stirring at 1000 rpm and kept there for 15 min. All these preparations were made in open air under laboratory conditions. The resulting geopolymer slurry was poured into sealed HDPE containers and cured at 80 °C for 2 days. The cured samples were kept at room temperature for 3 days after demoulding and placed in an oven at 80 °C for 2 days for drying. The dried samples were then ground in a pestle and mortar and sieved through a 100 μm sieve to obtain a powder with an average particle size of 20 μm with 3 μm and 90 μm D_10_ and D_90_ values, respectively.

### Silver incorporation into the geopolymer and thermal treatment

AgNO_3_ was dissolved in distilled water at different concentrations such that the amount of silver in the geopolymer powder would be 0.1, 0.5, 1.0, and 5.0% by weight, assuming complete incorporation of silver ions into the geopolymer powder. 30 g of the geopolymer powder was dispersed in 270 g of silver containing distilled water by a magnetic stirrer for 24 h at 50 °C. Then, the silver-corporated geopolymer powders were separated from water by vacuum filtration and dried under ambient conditions.

Chemical analysis of the silver-incorporated powders was carried out by X-ray Fluorescence (XRF, Rigaku ZSX Primus, Tokyo/Japan) using a semi-quantitative mode. Samples for XRF analysis were prepared by melting them using LiB_4_ at 10:1 LiB_4_:powder mass ratio, followed by casting them into glass tablets. The XRF results were reported as oxides present, except silver, in the samples without considering loss-on-ignition. The dried powders were calcined at 500 °C, 850 °C, and 1050 °C for 0.5 h. 10 °C/min heating rate and furnace cooling were used during the calcination experiments. These temperatures were chosen based on the fact that 500 °C represents the removal of the majority of water from the geopolymer structure, 850 °C represents the beginning of sintering, and 1050 °C represents the completion of sintering of the geopolymer powder. Phases present before and after the calcination were determined by X-ray diffraction (XRD, Rigaku Miniflex 600, Tokyo/Japan) using Cu Kα radiation at 40 kV and 15 mA with a scanning speed of 1° 2θ/min between 25° and 50° 2θ where silver-related compounds give their main reflections. Samples for the XRD measurements were prepared by pouring a small amount of powder into a glass sample holder’s cavity and pressing it gently to enough tightness to hold.

### Silver ion release behavior in water

The dried silver-incorporated geopolymer powders containing different amounts of silver were dispersed in fresh tap water at 3% (w/w) concentration and magnetically stirred for 24 h. The water was then filtered by a vacuum filter, and its silver ion content was measured by Inductively Coupled Plasma (ICP, Varian 720-ES-OES). To determine the sustained ion release behavior, the silver-incorporated powder with the highest silver content of 3.4% (w/w) was used. This powder was dispersed in fresh tap water at 3% (w/w) concentration, magnetically stirred for 1 h, and then separated from water by vacuum filtration. The amount of silver ions released into the water was measured by ICP. The same procedure was repeated five times using the same powder but fresh tap water each time. After each treatment, the amount of silver released into the water was measured.

### Characterisation studies

The thermal behavior of the geopolymer powder was characterized by simultaneous thermogravimetry and differential thermal analysis (TG–DTA, STA409 Netsczh, Selb/Germany) at a heating rate of 10 °C/min under flowing air. Dimensional changes occurring during the heating of the geopolymer were followed by optical dilatometry (Misura ODHT, Expert Systems, Sassuolo/Italy) of a pressed powder compact of the geopolymer powder. 10 °C/min heating rate was used up to 1000 °C during the test. The specific surface area and pore structure evolution before and after calcination were determined by the BET method (Nova Touch, LX1, Quantachrome, Boynton Beach, FL) using N_2_ gas. The samples were degassed at 200 °C for 6 h before the measurements. Microstructural examinations were performed by scanning electron microscopy (SEM, Zeiss Supra VP, Germany) using secondary electron imaging mode at an accelerating voltage of 20 kV and by transmission electron microscopy (TEM, JEOL 2100F, Tokyo/Japan) under bright field and high resolution (HRTEM) imaging conditions at an accelerating voltage of 200 kV. The SEM samples were prepared by sticking small fractured fragments on an aluminium stub using carbon tape, while the TEM samples were prepared by dispersing a small amount of powder in acetone, which was then applied on a copper grid. Samples for the SEM and TEM examinations were coated with a thin layer of gold and carbon, respectively, to prevent charging.

## Results and discussions

### Geopolymer characterization and thermal behavior

**Figure 1:**
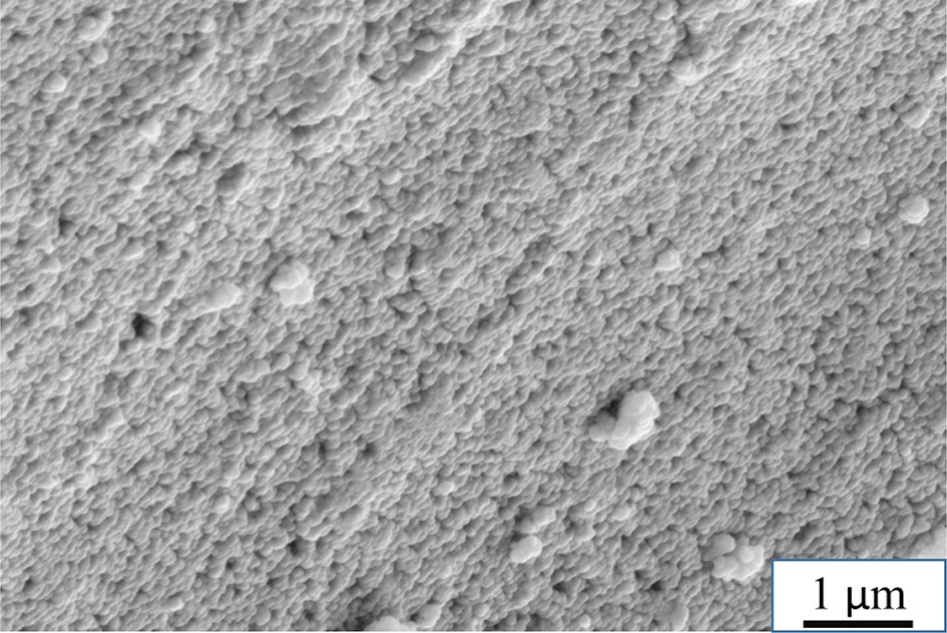
SEM secondary electron image of the as-synthesized geopolymer microstructure.

After setting at 80 °C, the geopolymer mix forms a rather hard and rigid body due to the geopolymerization reactions. Figure 1 shows an SEM image of the geopolymer microstructure showing networking of 50–100 nm size particles forming the geopolymer body. Between these nanoparticles, pores of approximately several tens of nanometers are present (see later Fig. 6a).

The geopolymer prepared in this work had pore sizes between 3 and 140 nm with an average pore size of 10.4 nm, as measured by nitrogen gas adsorption. Duxson^26^ found that the size of the pores in the geopolymer structure depends on the SiO_2_/Al_2_O_3_ molar ratio and measured pore sizes between 2.3 and 132 nm with an average value of 11.2 nm for the SiO_2_/Al_2_O_3_ molar ratio of 3.3, in agreement with this study. He also pointed out the presence of tiny pores within the particles (in the gel structure), some of which are not accessible even by nitrogen gas. Kriven et al.^34^ reported a similar pore structure of a geopolymer with the same SiO_2_/Al_2_O_3_ molar ratio, located between approximately 20 nm sized particles, and described the intrinsic microstructure of the geopolymer as a meshwork of nanoparticles separated by nanoporosity.

**Figure 2:**
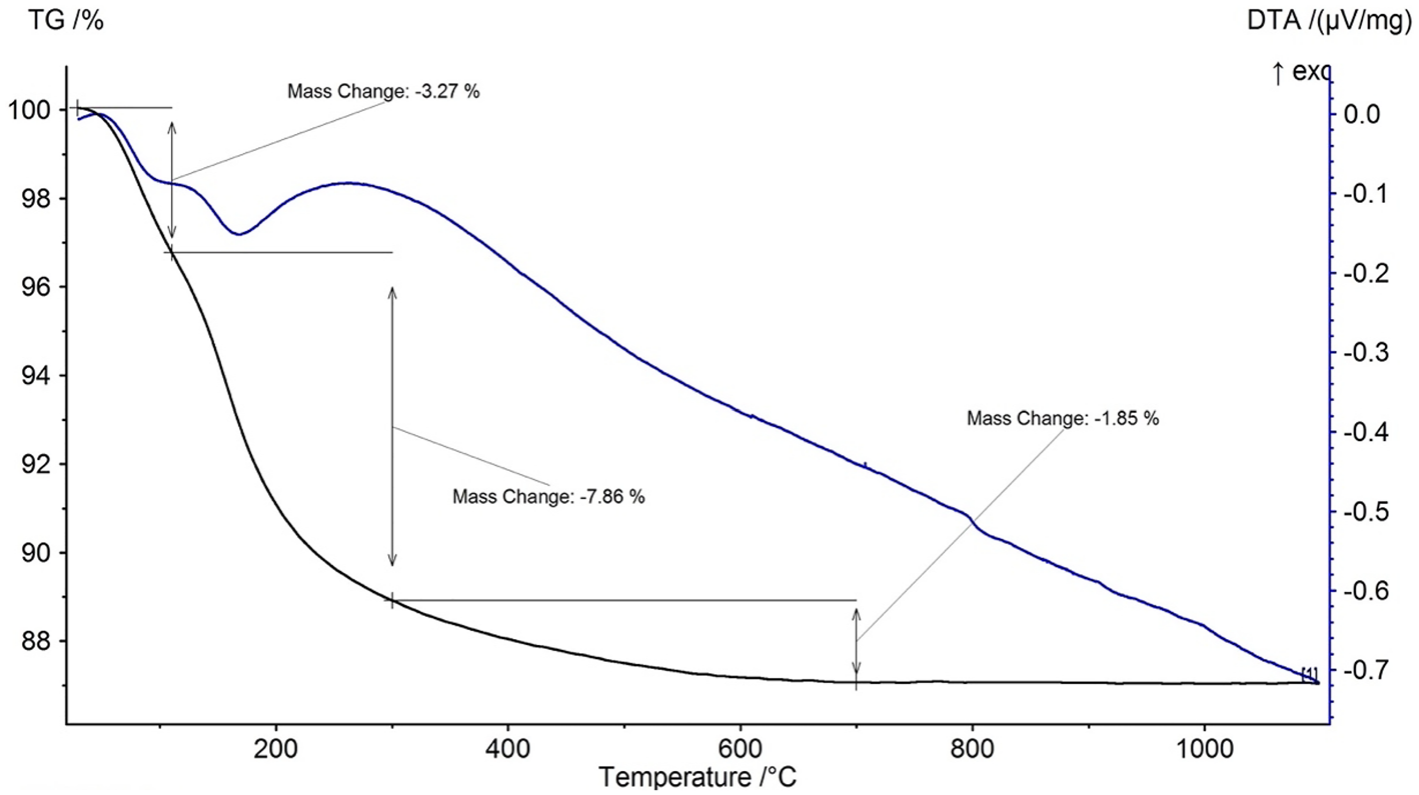
TG–DTA graph of the as-synthesized geopolymer.

The thermal behavior of the geopolymer powder is shown in Fig. 2. There are weight losses up to 700 °C, the majority of which occur below 300 °C. The weight loss below 100 °C is due to the removal of physical water from particle surfaces, and that seen between 100 and 300 °C could be ascribed to the evaporation of free water present in the nanopores^26,35^. These weight losses are associated with two clear endothermic peaks, seen at about 100 °C and 168 °C in the DTA graph. The weight loss continues up to 700 °C with a gradually decreasing rate and is ascribed to dehydroxylation caused by condensation of silanol and aluminol groups in the geopolymer structure^26^.

**Figure 3:**
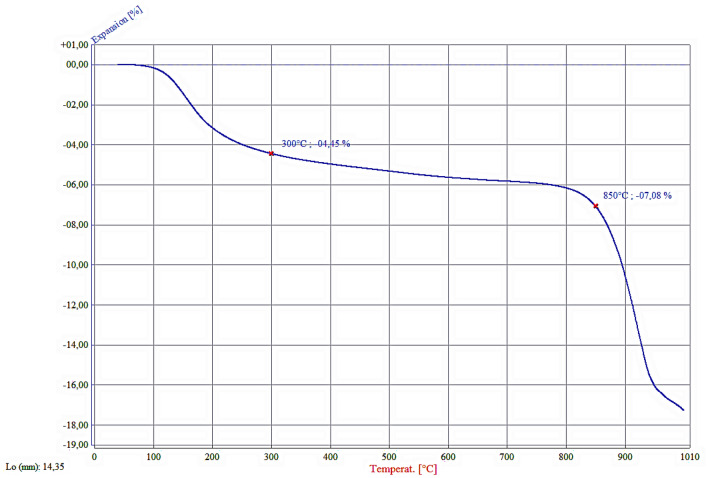
Shrinkage behavior of the as-synthesized geopolymer powder compact on heating measured by optical dilatometry.

Figure 3 illustrates the shrinkage behavior of the geopolymer powder compact during heating up to 1000 °C. There are two distinct regions with approximately 4.5% and 11.5% linear shrinkages between 100 and 300 °C and between 800 and 1000°C, respectively. A slow rate of shrinkage also occurs between 300 and 800 °C with corresponding 1.5% linear shrinkage. The shrinkages observed up to 800 °C correlate well with the weight loss behavior of the geopolymer shown in the TG graph in Fig. 2. Therefore, 4.5% shrinkage between 100 and 300 °C could be ascribed to capillary shrinkage caused by evaporation of free water from nanopores and about 1.5% shrinkage between 300 and 800 °C to the condensation of silanol and aluminol groups, as suggested by Duxson^26^. A large amount of shrinkage seen between 800 and 1000 °C is due to sintering and resultant porosity elimination^26,35^. These shrinkages in the geopolymer structure with temperature cause a reduction in the specific surface area, which is particularly substantial over 850 °C (Table 1). As seen in Table 1, the pure geopolymer powder had 22.5 m^2^/g, which was reduced to 19.5 m^2^/g and 12.2 m^2^/g after calcination at 500 °C and 850 °C, respectively.

**Table 1:** Changes in the specific surface area, pore volume, and average pore size of the geopolymer powder and 3.4% (w/w) silver-incorporated geopolymer powder upon calcination at various temperatures.

Calcination temperature	Specific surface area (m^2^/g)		Pore volume (cc/g)/average pore size (nm)	
	Pure geopolymer	Silver incorporated geopolymer	Pure geopolymer	Silver incorporated geopolymer
Uncalcined	22.5	18.5	0.054/10.4	0.026/6.8
500 °C	19.5	14.9	0.055/12.1	0.030/9
850 °C	12.2	9.2	0.03/11	0.02/9.6
1050 °C	-	2.0	-	-

### Silver incorporation and calcination

Table 2 gives the chemical analysis of the geopolymer powder and the silver-incorporated geopolymer powders based on the oxides present, except silver. The targeted amount of silver incorporation is also included in the table. The impurity elements coming from metakaolin were not shown for the sake of clarity in the analysis. As shown in Table 2, somewhat less amount of silver than the targeted was incorporated into the geopolymer structure at 1% (w/w) and 5% (w/w) targeted values, being 0.7% (w/w) and 3.4% (w/w), respectively. This may be due to the rather longer treatment time (24 h) in the solution, which may cause the release of Ag^+^ back into the solution at high Ag^+^ loading levels. The reduction in Na_2_O content in the silver-incorporated sample suggests that the exchange of Na^+^ with H^+^ ions from water also occurs during the silver incorporation process.

**Table 2:** Chemical compositions of geopolymer powders after silver incorporation. Note that silver is reported as pure silver for easy comparison with the targeted amount.

Ag% (targeted)	Ag (achieved)	SiO_2_	Al_2_O_3_	Na_2_O
–	–	53.76	30.85	14.07
0.1%	0.09	53.98	33.40	10.97
0.5%	0.51	53.01	33.58	11.38
1.0%	0.70	54.62	32.43	10.74
5.0%	3.40	52.84	32.35	9.96

**Figure 4:**
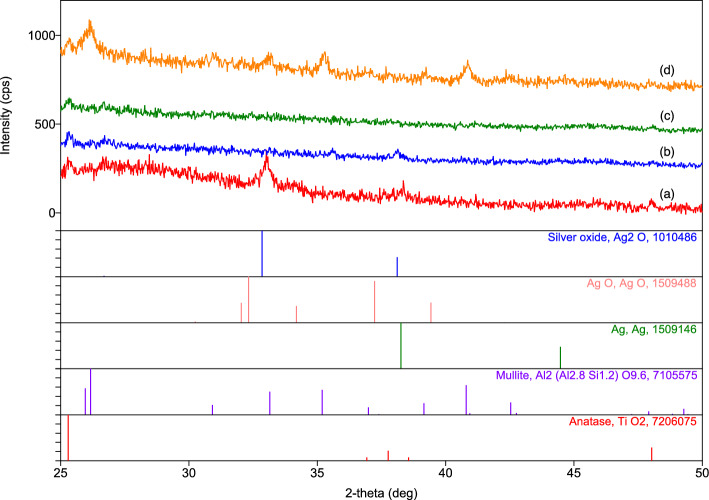
X-ray diffraction spectra of 3.4% (w/w) silver-containing geopolymer powder after (**a**) synthesis and after calcination at (**b**) 500 °C, (**c**) 850 °C, and (**d**) 1050 °C.

Figure 4 shows the XRD spectra of 3.4% (w/w) silver-incorporated samples after the synthesis (uncalcined) and after calcination at different temperatures. In the as-synthesized form, apart from anatase (TiO_2_), which is an impurity phase coming from the metakaolin and also persists in the calcined samples, the presence of Ag_2_O is evident (Fig. 4a). This indicates that under the given experimental conditions, silver precipitates according to the following reaction:

$$ {\text{2AgNO}}_{{{3}({\text{aq}})}} + {\text{ 2NaOH}}_{{({\text{aq}})}} \to {\text{ Ag}}_{{2}} {\text{O}}_{{({\text{s}})}} + {\text{ 2NaNO}}_{{{3}({\text{aq}})}} + {\text{ H}}_{{2}} {\text{O}}_{{({\text{aq}})}} $$

The pH of the solution containing 3% (w/w) of the dried geopolymer powder was 11, and the above reaction occurs easily above pH 9^29^. After calcination at 500 °C, the additional minor peaks seen at 35.6 and 38.2 degrees 2θ do not match with any of the silver compounds (Fig. 4b) and are unidentified. The powder calcined at 850 °C gave an amorphous spectrum (Fig. 4c) and that calcined at 1050 °C contained only a minor amount of mullite, which was probably due to the presence of unreacted metakaolin in the geopolymer, which converted into mullite (Fig. 4d).

**Figure 5:**
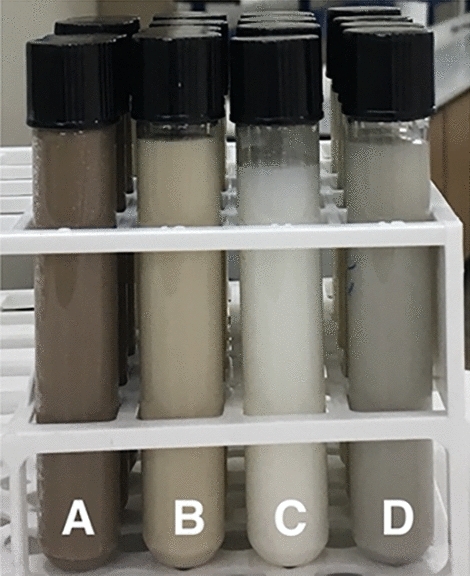
Colors of 3% (w/w) suspensions of 3.4% (w/w) silver-incorporated geopolymer powders. A, B, C, and D denote suspensions of the powders after synthesis and after calcination at 550 °C, 850 °C, and 1050 °C, respectively.

Figure 5 shows the colors of 3% (w/w) suspensions of 3.4% (w/w) silver-incorporated geopolymer powder before and after the calcination. The brown color of the suspension containing the as-synthesized powder is due to Ag_2_O, which has a brown color itself^36^. However, the color of the suspension changes to creamy yellow and white for 500 °C and 850 °C calcined powders, respectively, and does not represent any characteristic colors of silver or its oxide compounds. The gray color of the suspension containing 1050 °C calcined sample is the characteristic colour of AgO^36^, although the presence of AgO was undetectable at this temperature in the XRD spectrum (Fig. 4d). These observations indicate that some structural changes occur in the precipitated silver oxide compound during calcination.

Concerning the surface area development after 3.4% (w/w) silver incorporation, silver incorporation caused a slight reduction in the surface area from 22.5 to 18.5 m^2^/g (Table 1), probably due to the filling of some pores by Ag_2_O precipitates. The reduction in pore volume with the silver incorporation from 0.054 cc/g to 0.026 cc/g (Table 1) supports this explanation. Reduction in the specific surface area due to the calcinations followed a similar trend to the pure geopolymer, as one would expect, in that 500 °C, 850 °C, and 1050 °C calcined samples had 14.9 m^2^/g, 9.2 m^2^/g, and 2.0 m^2^/g specific surface areas, respectively.

**Figure 6:**
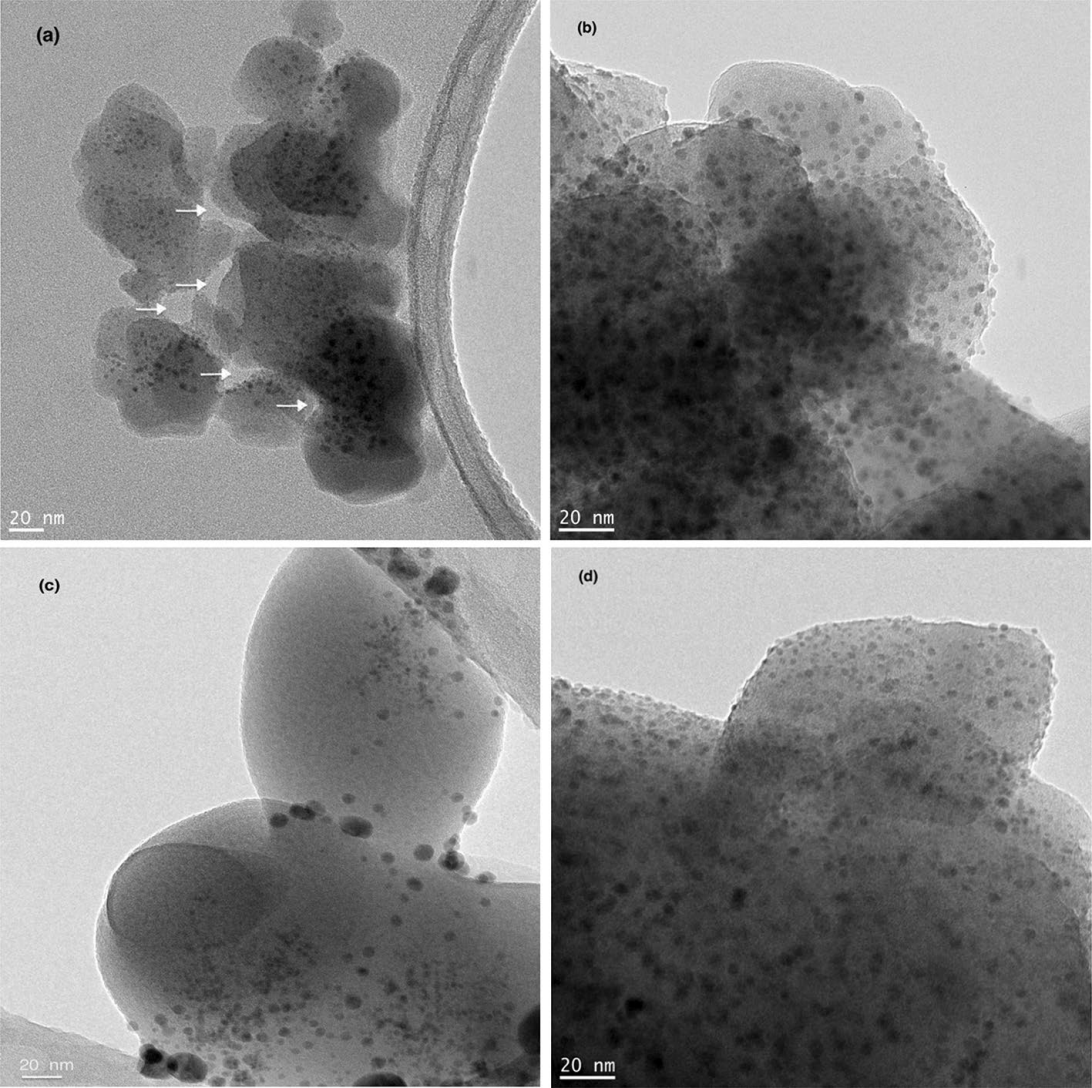
Bright-field TEM images of 3.4% (w/w) silver-incorporated geopolymer powder: (**a**) and (**b**) as-synthesized, (**c**) after calcination at 850 °C and (**d**) after calcination at 1050 °C. Arrows in (**a**) indicate pores between the geopolymer nanoparticles. Silver oxide precipitates appear dark in the images due to their higher atomic number related to electron absorption.

Further examinations of the silver oxide precipitates were carried out by TEM to observe their size, morphology, and structural development during calcination. Figure 6a,b show bright-field TEM images of 3.4% (w/w) silver-incorporated geopolymer powder after the synthesis, while Fig. 6c,d show those after the calcination at 850 °C and 1050 °C, respectively. The lower magnification TEM image (Fig. 6a) shows the geopolymer particles as well as the pores between them (arrowed). The fine dark features within the particles are Ag_2_O precipitates, as confirmed by XRD in Fig. 4a. It should be noted that Ag_2_O predominantly precipitates in small pores within the geopolymer particles, not in the larger pores (arrowed in Fig. 6a) between them. This suggests that smaller pores present preferential sites for precipitation. Higher magnification images in Fig. 6b show that the sizes of the Ag_2_O precipitates are extremely fine, ranging between 2 and 5 nm with occasional larger (10–20 nm) ones. Interestingly, their size remains stable upon calcination up to 1050 °C, as seen in Fig. 6c, d, despite a large amount of densification-related shrinkage at this temperature (Fig. 3). It should be noted that such a fine precipitate size may be the reason behind the absence of the diffraction peaks related to the silver compounds in the XRD spectra in Fig. 4 in that excessive broadening caused a few nanometer-sized crystals^37^ as well as their low concentration may result in lower intensities, which may be masked by the background spectra.

**Figure 7:**
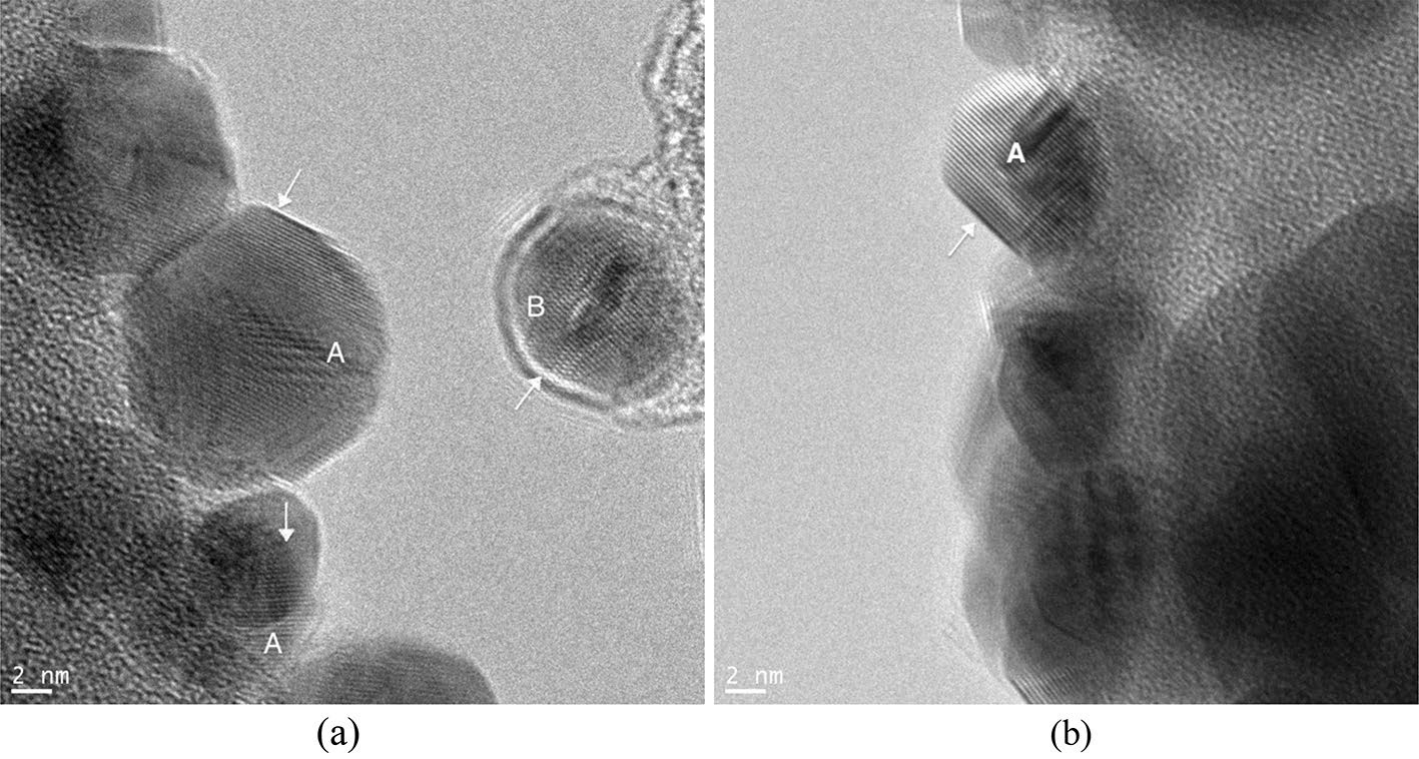
HRTEM images of silver oxide precipitates in samples calcined at (**a**) 850 °C and (**b**) 1050 °C.

Indeed, HRTEM images of 850 °C and 1050 °C calcined samples are shown respectively in Fig. 7a,b support this explanation by showing the presence of crystalline precipitates. Lattice spacing in the direction of the arrow at particle A and particle B indicated in Fig. 7a were measured to be 1.93 Å and 2.36 Å which corresponds to (d_211_) and (d_200_) of Ag_2_O, respectively, and that in the direction of the arrow at the particle A indicated in Fig. 7b was measured to be 2.28 Å which corresponds to ${({\text{d}}}_{20\overline{2} })$ of AgO. While the gray colour of the 1050 °C calcined sample’s suspension in Fig. 5 supports the presence of AgO, the white colour of the 850 °C calcined sample’s suspension does not represent the characteristic colour of Ag_2_O^36^. It is to be noted that the HRTEM examinations were limited to a few precipitates and did not represent the whole sample.

### Silver ion release behavior

**Figure 8:**
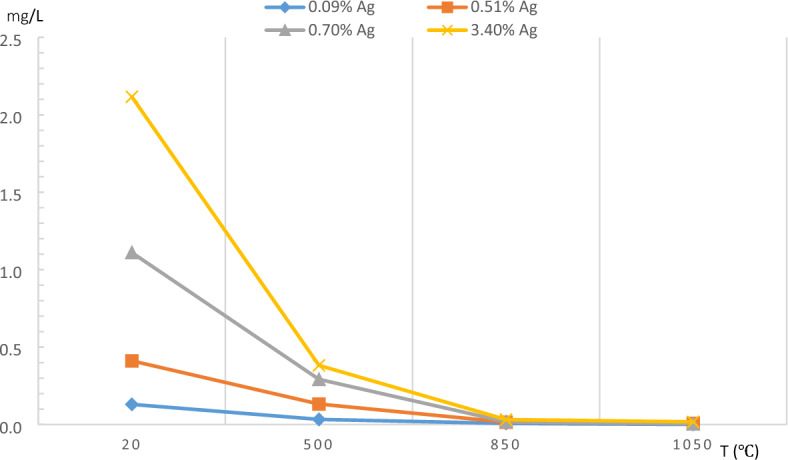
Effect of calcination temperature on Ag^+^ release behavior for different amounts of silver-containing geopolymer powders after immersion in tap water for 24 h at 3% (w/w) concentration.

Figure 8 shows Ag^+^ release behavior before and after the calcinations of the geopolymer powders incorporated with different amounts of silver when soaked in tap water for 24 h. The Ag^+^ release values were also given as tabulated in Table 3 for clarity. It is seen that the Ag^+^ release behavior of the geopolymer powders is reduced substantially by increased calcination temperature and reaches very low levels after calcination at 1050 °C. It can also be deduced from Fig. 8 and Table 3 that the amount of silver ion released increases with increasing amount of silver incorporation in the geopolymer powder, as expected due to the availability of a higher number of the precipitates for dissolution.

**Table 3:** Effect of calcination temperature on Ag^+^ release behavior for different amounts of silver-containing geopolymer powders after immersion in tap water for 24 h at 3% (w/w) concentration.

Ag content of the geopolymer %	20 °C (mg/L)	500 °C (mg/L)	850 °C (mg/L)	1050 °C (mg/L)
0.09%	0.1304	0.0333	0.0074	0.0006
0.51%	0.4114	0.1326	0.0155	0.0075
0.70%	1.1103	0.2920	0.0179	0.0042
3.40%	2.1160	0.3822	0.0322	0.0177

The decreased Ag^+^ release behavior with the increased calcination temperatures could be explained by the thermal behavior of the geopolymer. As it was shown in Fig. 3, the geopolymer powder compact experiences shrinkages during heating and the reasons for this have been discussed previously. As a result of these shrinkages, the specific surface area decreases such that about 50% of the surface area is lost after calcination at 850 °C with a concurrent reduction in the number of nanopores (Table 1) due to partial sintering since the sintering starts at 800 °C. At 1050 °C, the densification, evidenced by a large amount of shrinkage, removes the majority of the pores and further reduces the specific surface area to 2 m^2^/g, representing about 90% reduction. These results demonstrate that the rather previous structure of the geopolymer becomes progressively tightened with increasing calcination temperature. This, in turn, makes Ag^+^ diffusion from the pores into the solution increasingly difficult and explains the reason for the reduced ion dissolution seen in Fig. 8. It should be noted that the solubilities of Ag_2_O and AgO in water are similar, being 0.025 g/L and 0.027 g/L at 25 °C^38^ and even similar Ag^+^ release in water with natural pH was reported from a geopolymer matrix containing silver nanoparticles, colloidal silver and, Ag_2_O precipitates^29^. Therefore, the crystalline structure of the silver oxide precipitates needs not to be considered as a parameter for Ag^+^ dissolution as well as the size of the precipitates since it remains constant with temperature (Fig. 6). These results show that by simply choosing an appropriate calcination temperature, silver ion release can be tuned as desired. That is, some applications may require fast silver ion release, such as in medical applications^20^, but others may require a sustained and repeatable release of Ag^+^ that is effective but at the same time non-hazardous to the environment, such as in water filtration. For instance, the presence of approximately several tens of µg/L Ag^+^ is reported to be sufficient for antimicrobial activity in water^4^ which is below the WHO recommended maximum contaminant level limit (100 µg/L) for drinking water ^39^.

Another important aspect of materials for antibacterial applications is the longevity of the antibacterial effect, which could be assured by a repeatable and sustained Ag^+^ release behavior^4^. Table 4 shows the amount of Ag^+^ released from the 3.4% (w/w) silver incorporated geopolymer powder after the synthesis and after calcination at 850 °C when they are kept repeatedly in fresh tap water for 1 h each time under stirring at 3% (w/w) concentration. The initial higher Ag^+^ release observed in the first treatment sequence in both samples may be related to loosely bound Ag^+^ on the geopolymer powder surfaces and/or in its pores. Although the reduction in Ag^+^ release between the 2nd and 5th cycle in the uncalcined sample reaches up to 30% (compare 2.177 mg/L vs 1.321 mg/L), that in the 850 °C calcined sample is less than 10% (compare 0.061 mg/L vs 0.056 mg/L). Nevertheless, the table clearly shows that the silver-doped geopolymer has a repeatable and sustained Ag^+^ release behavior, and this property is also preserved upon calcination.

**Table 4:** The amount of Ag^+^ released from 3.4% (w/w) silver-containing geopolymer powder when it is repeatedly immersed in fresh tap water for 1 h at 3% (w/w) concentration.

Immersion sequence	Uncalcined (mg/L)	850 °C calcined (mg/L)
1	5.381	0.103
2	2.177	0.061
3	1.638	0.060
4	1.594	0.057
5	1.321	0.056

## Conclusion

In this study, different amounts of silver-containing geopolymers were prepared, and their Ag^+^ release behavior was studied after calcination at various temperatures and correlated with the thermal behavior of the geopolymer.

The geopolymer microstructure is composed of a network of nanoparticles with nanoporosity located between and within the particles. When the geopolymer powder is dispersed in an AgNO_3_-containing solution, silver is precipitated as Ag_2_O with mainly about 2–5 nm size, particularly in the fine pores within the nanoparticles. The size of the precipitates remains stable even after complete densification of the geopolymer at 1050 °C. The silver oxide-incorporated geopolymer shows repeated and sustained Ag^+^ release behavior. The amount of Ag^+^ released is reduced by the calcination temperature, but the sustained release property is maintained. These findings suggest that silver-containing geopolymers could be a viable material for tunable Ag^+^ supply in a sustained manner for various antibacterial applications. While 3.4% (w/w) silver doped geopolymer in the uncalcined state could find applications in medical fields due to its high amount of Ag^+^ release, its 800 °C calcined form could be used in water disinfection applications as its Ag^+^ release amount is below the maximum contaminant level limit (100 µg/L) for drinking water. With this respect, as a future work, it would be interesting to study Ag^+^ release rates also in a dynamic flow-through system.

## Data Availability

All data generated or analyzed during this study are included in this published article. Datasets are available in the manuscript.
